# Effect of cofactors on NAFLD/NASH and MAFLD. A paradigm illustrating the pathomechanics of organ dysfunction

**DOI:** 10.20517/mtod.2022.14

**Published:** 2022-08-22

**Authors:** Amedeo Lonardo, Ashwani K. Singal, Natalia Osna, Kusum K. Kharbanda

**Affiliations:** 1 Department of Internal Medicine, Azienda Ospedaliero-Universitaria, Modena 41126, Italy.; 2 Sanford School of Medicine, University of South Dakota, Vermillion, SD 57105, USA.; 3 Research Service, Veterans Affairs Nebraska-Western Iowa Health Care System, Omaha, NE 68105, USA.; 4 Department of Internal Medicine, University of Nebraska Medical Center, Omaha, NE 68198, USA.; 5 Department of Biochemistry & Molecular Biology, University of Nebraska Medical Center, Omaha, NE 68198, USA.

**Keywords:** Alcohol, diet, HBV, HCV, HIV, infection, immunity, MAFLD, NAFLD, NASH, metabolic syndrome, HCC, maternal obesity, microbiota, personalized medicine, physical activity, sex differences

## Abstract

Primary nonalcoholic fatty liver disease (NAFLD) is bi-directionally associated with the metabolic syndrome and its constitutive features (“factors”: impaired glucose disposal, visceral obesity, arterial hypertension, and dyslipidemia). Secondary NAFLD occurs due to endocrinologic disturbances or other cofactors. This nosography tends to be outdated by the novel definition of metabolic associated fatty liver disease (MAFLD). Irrespective of nomenclature, this condition exhibits a remarkable pathogenic heterogeneity with unpredictable clinical outcomes which are heavily influenced by liver histology changes. Genetics and epigenetics, lifestyle habits [including diet and physical (in)activity] and immunity/infection appear to be major cofactors that modulate NAFLD/MAFLD outcomes, including organ dysfunction owing to liver cirrhosis and hepatocellular carcinoma, type 2 diabetes, chronic kidney disease, heart failure, and sarcopenia. The identification of cofactors for organ dysfunction that may help understand disease heterogeneity and reliably support inherently personalized medicine approaches is a research priority, thus paving the way for innovative treatment strategies.

## DEFINITIONS AND BURDEN

Nonalcoholic fatty liver disease (NAFLD) is an umbrella definition encompassing the clinico-pathological spectrum of disorders spanning from simple steatosis to nonalcoholic steatohepatitis (NASH), with or without fibrosis, cirrhosis, and hepatocellular carcinoma (HCC)^[1,2]^. This implies that NAFLD defines a gamut of conditions mimicking alcohol-related liver disease but are seen in patients without alcohol use disorder^[3]^. In principle, in addition to alcohol, other competing causes of liver disease should be ruled out in the NAFLD field, notably including HCV infection and thyroid disorders, although the extent to which alternative etiologies need to be excluded remains poorly defined^[4]^. Moreover, the rationale for distinguishing alcohol-related liver disease from NAFLD may sometimes appear uncertain^[5,6]^.

Clinically and epidemiologically, NAFLD is important given that it exacts a heavy toll in terms of patient quality of life^[7]^ and, owing to direct and indirect costs, accounts for substantial healthcare expenditures^[8–10]^.

## CLASSIFICATION

Based on its pathogenic framework, NAFLD may be categorized as either primary or secondary disease. *Primary NAFLD* exhibits a mutual and bi-directional association with the metabolic syndrome and its individual components: impaired glucose disposal, visceral obesity, atherogenic dyslipidemia, and arterial hypertension^[11]^. These *“factors”* tend to cluster, such that the appearance of each predicts the future development of others^[12]^. However, there are also several *secondary NAFLD forms*^[13]^. These secondary NAFLD forms may, in their turn, be classified as illustrated in Table 1^[13–26]^.

Although the topic remains open for discussion, notions reported in Table 1 suggest that the most common secondary NAFLD forms occur in the setting of specific endocrine derangements and inherited metabolic disorders.

## NAFLD *VS*. MAFLD - LIMITATIONS OF LIVER BIOPSY

The proposal to rename NAFLD to metabolic-associated fatty liver disease (MAFLD)^[27,28]^, which has met a universally favorable reception^[16,29]^, appears to be a logical attempt to overcome the two principal limitations and inconsistencies inherent in the NAFLD/NASH definition (discussed below) while emphasizing the association of hepatic fatty changes with the metabolic syndrome and its components^[30]^. The main drawbacks of the NAFLD nosography include: (a) liver biopsy; and (b) exclusion of alcohol consumption. (a) The practice of liver biopsy in NAFLD arena must be considered with a prudent and balanced view. On the one hand, NASH is a clinico-pathological disease entity that, by its very definition, requests histological documentation^[31]^. On the other hand, liver biopsy is invasive, not painless, nor devoid of stress for the patient; it may carry risky complications such as bleeding and perforation of hollow organs; and may even be (rarely) mortal^[32–34]^. Moreover, liver histology changes may be patchily distributed through the hepatic parenchyma opening to disease misclassification from sampling error^[35,36]^, and it is fibrosis (which can also be assessed non-invasively), not NASH, that dictates the prognosis in NAFLD^[37]^. Finally, it is uncertain whether - for clinical practice purposes - we do have to perform an invasive and potentially risky procedure without prescribing any approved drugs or biological treatments for NASH or related liver diseases to our patients^[34,38]^. All the above perplexities may be overcome by the less committal diagnosis of MAFLD, which does not request liver biopsy^[39]^. (b) There are no objective and reliable biomarkers of alcohol consumption to define whether a given liver disease is “alcoholic” or “nonalcoholic”^[40]^. Unless stated otherwise, it is difficult to objectively determine the number of alcoholic units consumed or, alternatively, the duration of alcohol abstinence. At the same time, the rationale for separating alcohol-related from nonalcoholic liver disease has also been deemed to be questionable based on histological and pathogenic grounds^[41,42]^. Collectively, those arguments summarized above under Points (a) and (b) further reinforce the rationale for transitioning from NAFLD to MAFLD.

## NAFLD PATHOGENIC HETEROGENEITY AND HISTOLOGICAL BASES OF CLINICAL VARIABILITY

NAFLD exhibits some prominent features that uniquely characterize its pathophysiological and clinical profile. First, it is a systemic disorder whose manifestations reach far beyond the liver^[43,44]^. Second, it has a remarkable pathogenic heterogeneity and runs an unpredictable course in the individual patient^[45]^. Third, it has a distinct sexual dimorphism^[46,47]^. Ideally, it would be tempting to speculate that it is pathogenic heterogeneity, including the impact of sex and reproductive status, that will eventually dictate a natural course in any given patient. Although this notion is reasonable, we are still far from having clear evidence for this conclusion. What is certain is that the course of NAFLD exhibits a remarkable variety of target organ dysfunction^[48]^. This spans from the liver (cirrhosis and HCC) (2), pancreatic beta-cell (diabetes)^[49]^, the kidneys (chronic kidney disease)^[50]^, the skeletal (sarcopenia), cardiac muscles (heart failure)^[51,52]^, and the lungs (impaired function)^[53,54]^ to the development of cancer in a variety of organs^[55,56]^. What then determines such an impressively diverse clinical course in the individual subject?

In 2018, Vilar Gomez *et al*. published a breakthrough study in the area, identifying liver histology as a determinant of hepatic versus extrahepatic disease manifestations. With a 5.5-year follow-up on a cohort of approximately 460 biopsy-proven NAFLD patients, they found that cirrhosis was associated with predominantly liver-related events, while bridging fibrosis was linked to the development of predominantly non-hepatic cancers and vascular events^[57]^. Although it would be unimaginable for all patients with NAFLD to undergo a liver biopsy, it is anticipated that either non-invasive biomarkers of fibrosis or imaging techniques quantifying fibrosis may serve as a substitution for liver biopsy in determining the course of disease^[58,59]^. Again, although perfectly plausible, this hypothesis remains to be tested in further prospective studies.

**Figure 1.**
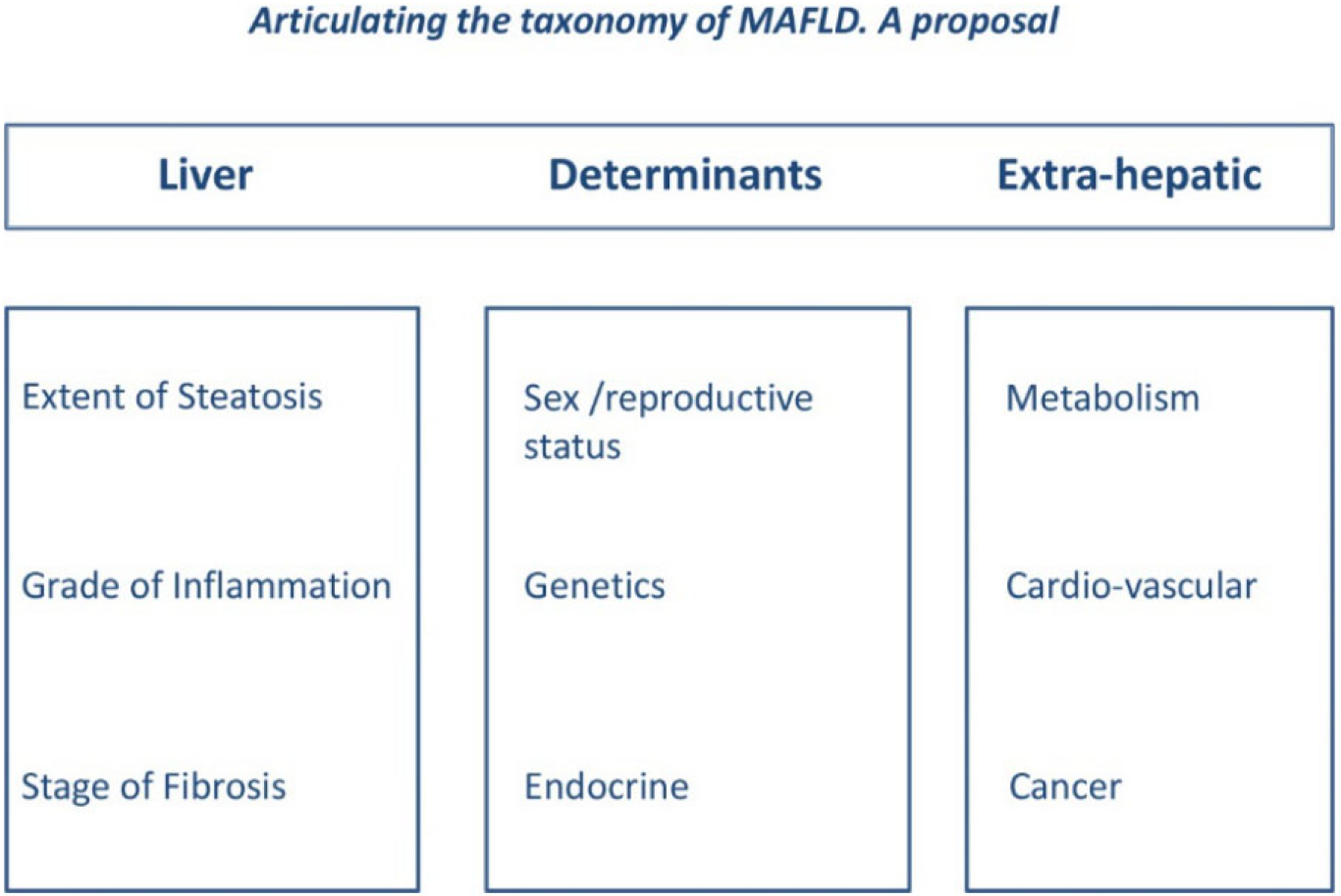
The LDE system (reprinted from^[4]^). The LDE system, which may be applied to both NAFLD and MAFLD, exhibits a basic syntax including a prefix (“L” for liver), a pathogenic core (“D” for determinants), and a suffix (“E” for extrahepatic). Liver (L): Information regarding liver health, which may also be obtained non-invasively other than histologically. Determinants (D): Information including sex and reproductive status, genetic determinants, and (minimal) endocrine assessment. Extrahepatic (E): Data on extrahepatic manifestations of disease. For example, illustrating this proposed classification, patient Mr. Max Green might be declared to have MAFLD/NAFLD (L, steatosis mild, inflammation absent, and fibrosis absent; D, hypothyroid, no SNP identified, and associated with full-blown MetS; and E, arterial hypertension, medio-intimal carotid thickening, and previous colon cancer).

The selection of more homogenous patient populations with more predictable disease outcomes, and presumably higher treatment response rates, represents a research priority due to the disappointing results of many NASH trials^[60]^. While, in the future, a precise metabolic identity card may best characterize the individual NAFLD patient^[61]^, this tool is not yet available in clinical practice. A feasible strategy for this goal could be identifying similar phenotypic subgroups. Given the systemic nature of NAFLD, a simple classification system should include liver, pathogenic determinants, and extrahepatic (LDE) features, as illustrated in Figure 1.

## NAFLD COFACTORS

There is no unified definition of “cofactors” in the NAFLD/MAFLD field, although this term was extensively evaluated and studied in the HCV arena in the past^[62]^. In this perspective, we define “cofactors”, such as clinically relevant disease modifiers, as “cofactors” that interact with “metabolic factors” in the field of metabolic syndrome.

Interestingly, these cofactors may have diagnostic implications (e.g., genetics), and some are modifiable (e.g., lifestyle habits and infection). These cofactors are innumerable, and the current perspective does not aim to be exhaustive on the cofactor spectrum. Instead, some of the best-characterized examples of NAFLD cofactors are discussed below. Emphasis is given to those that have been better characterized, are more extensively evaluated, or appear to be more promising.

As illustrated in our Graphical Abstract, the current perspective has five sections: (1) genetics and epigenetics; (2) drinking and eating habits; (3) sedentary behavior; (4) immunity and drugs; and (5) viral infections.

### Genetics and epigenetics

Studies demonstrating that first-degree relatives of NAFLD patients exhibit a much higher risk of the disease compared to the general population support the notion that genetics and epigenetics play a key role in the development of NAFLD^[63]^. Indeed, genome-wide association studies have identified numerous genetic polymorphisms involved in NAFLD development and progression, e.g., patatin-like phospholipase domain-containing protein 3 (PNPLA3), membrane-bound O-acyltransferase domain containing 7 (MBOAT7), transmembrane 6 superfamily member 2 (TM6SF2), glucokinase regulator (GCKR), and others^[64,65]^. However, whether “metabolic NAFLD” (i.e., MAFLD) and “genetic NAFLD” follow the same natural course remains unproven^[66,67]^. PNPLA3 variant rs739409: C > G on chromosome 22 is the single most replicated variant in liver diseases and was first identified in 2008 in association with NAFLD^[68]^. It has now been firmly established as a gene modifier of hepatic steatosis and a risk factor for liver disease progression^[69]^. It is a non-synonymous single nucleotide mutation altering a highly conserved amino acid isoleucine to methionine at residue 148. PNPLA3 encodes for adiponutrin, a transmembrane protein that has lipogenic transacetylase and triglyceride hydrolase activities. It is suspected that I148M promotes hepatic intracellular lipid accumulation by reducing the breakdown of triglycerides stored in the lipid droplets^[70]^. A non-synonymous single nucleotide variant in the TM6SF2 (rs58542926: C > T (E167K) on chromosome 19 is associated with hepatic triglyceride content and is an independent risk factor for liver fibrosis and HCC^[71]^. Recent studies demonstrate that TM6SF2 acts in the smooth endoplasmic reticulum to promote bulk lipidation of apolipoprotein B-containing lipoproteins, thus preventing fatty liver disease^[72]^. MBOAT7 encodes an enzyme with lysophosphatidylinositol acyltransferase activity, and its variant, rs641738 C > T, is associated with NAFLD^[73]^ and fibrosis in patients with a BMI < 35 independent of lobular inflammation^[74]^. Importantly, in animal models, its loss of function is sufficient to promote NAFLD progression^[75]^. Glucokinase regulator (GCKR) encodes glucokinase regulatory protein (GKRP), a hepatocyte-specific inhibitor of the glucose-metabolizing enzyme glucokinase, a primary glucose sensor^[76]^.

Epigenetic mechanisms, comprising histone methylation, abnormal DNA methylation, and circulating miRNA profiles, all interact with inherited risk factors to determine individual susceptibility to NAFLD and, compared to genetic mechanisms, are affected by the patient’s lifestyle changes^[64,77,78]^. The finding that adaptions to maternal obesity in early life increase the susceptibility to developing NAFLD and its complications in offspring^[79]^ is an excellent example of the role of epigenetic factors in NAFLD pathobiology. Hagström *et al*., in their population-based study recruiting 125 biopsy-proven cases compared to 717 controls, consistently found that maternal BMI early in pregnancy was an independent risk factor for the diagnosis and severity of NAFLD in their offspring (OR in offspring to obese mothers: 3.26, CI 1.72–6.19, for any NAFLD and 3.67, CI 1.61–8.38, for fibrotic NAFLD)^[80]^. This study indirectly suggests that educational campaigns aimed at improving diet and encouraging physical exercise would reduce the risk of obesity-related conditions in mothers and their offspring and should be conducted among obese women of fertile age^[80]^. Interestingly, evaluation of liver transcriptome profiles in rats has shown that maternal obesity programs sex-dependent changes in offspring hepatic gene expression leading to more severe insulin resistance and NAFLD among male offspring than female counterparts^[81]^. Moreover, by comparing germ-free mice colonized with stool microbes from two-week-old infants born to either obese or normal-weight mothers, Soderborg *et al*. demonstrated that altered gut microbiome composition (i.e., dysbiosis) results in increased hepatic inflammatory responses and triggers NAFLD and excess weight gain in germ-free mice colonized with stool microbes from two-week-old infants born to obese mothers^[82]^.

Together, genetic and epigenetic cofactors participate in NAFLD development and progression and carry translational implications, which can be exploited to implement personalized medicine approaches^[64,83]^. These include programs for targeted screening and surveillance of complications, prediction of the individual response to pharmacological therapies, and opportunities for using miRNAs for treating liver disease and utilizing the gene variant as the therapeutic target^[78,84]^. Lifestyle habits predisposing to the development and progression of NAFLD represent a holistic scenario including sedentary behavior and unhealthy dietary patterns, which are discussed below under Points 2 and 3.

### Eating habits

If the Mediterranean diet (Med-diet), featuring homemade, unprocessed plant-based foods as well as fish and poultry in low to moderate amounts, is deemed to protect from NAFLD and NASH, the growing global consumption of ultra-processed hypercaloric foods enriched in simple sugars and hydrogenated fats is deemed to facilitate the metabolic syndrome, steatosis, and its histological progression^[85]^. These notions have tremendous clinical potential in as much as they indicate what NAFLD/NASH/MAFLD patients should be suggested to eat^[86]^. For example, a meta-analysis and meta-regression analysis of six randomized controlled trials found that - compared to the control diet - Med-Diet was associated with significant reductions of fatty liver index (FLI) and homeostasis model assessment of insulin resistance (HOMA-IR), suggesting that Med-Diet is a beneficial pharmaco-nutritional therapy in NAFLD^[87]^.

Additionally, recent studies refute the classic notion that moderate alcohol consumption might be beneficial in NAFLD; thus, alcohol should best be avoided per guideline recommendations (reviewed in^[87]^). Concerns about the potential linear dose-response on the pro-fibrogenic and carcinogenic effect of alcohol^[88–92]^ fully support the notion that alcohol is a cofactor that potentially causes target organ dysfunction^[93–95]^. Dietary habits are inextricably connected with physical activity patterns.

### Physical (in) activity

Studies have shown that NAFLD, physical inactivity and depressive symptoms form a dangerous pathogenic triangle^[4,96,97]^. Weinstein *et al*. provided proof-of-concept of this notion by analyzing the Rancho Bernardo Study of Healthy Aging. Overall, 589 individuals were included in the analyses. Data show that individuals with NAFLD have high levels of physical inactivity, particularly those with depressive symptoms^[98]^. Of concern, a low level of physical activity, in turn, is associated both with an increased NAFLD prevalence and with unfavorable cardio-metabolic and hepatic outcomes of NAFLD^[99,100]^. Thus, increasing physical activity remains an undisputed mainstay for preventing and managing NAFLD and related organ dysfunction. Additionally, lifestyle habits are known to be associated with immunity patterns.

### Infection, immunity, and microbiota

While NAFLD is deemed to predispose to a variety of infections, including bacterial^[101]^, the impact of infection on NAFLD course has mostly focused on viral infections. Probably the earliest and best-characterized examples include viral hepatitis C and B^[15,102,103]^. Additionally, many data address the deleterious interaction of NAFLD with HIV infection^[104,105]^. More recently, researchers have focused on SARS-CoV-2 infection^[106–108]^. Collectively, data suggest that viral infections are strongly associated with NAFLD outcomes, implying their role as disease cofactors. Immune dysfunction is a vast and under-appreciated aspect that likely plays a wide range of pathogenic roles spanning from NAFLD pathobiology and progression^[109,110]^ and the interaction of NAFLD with autoimmune (liver) disorders^[111–113]^ to drug-induced liver injury occurring in NAFLD individuals^[114,115]^. Whether and which tests exploring immune dysfunction in NAFLD should be used to better characterize NAFLD phenotypes remains to be defined.

Gut microbes comprise bacteria, fungi, viruses, archaea, and protozoa. The bacterial microbiome in healthy humans is dominated by beneficial bacterial phyla such as Bacteroides and Firmicutes, and a smaller proportion consists of Proteobacteria, Actinobacteria, and Verrucomicrobia^[116]^. The gut bacterial microbiome in patients with liver disease is characterized by dysbiosis with an increase in harmful and a decrease in beneficial bacteria, and this abnormality worsens with increased disease severity and is also associated with liver and patient-related outcomes^[117]^.

Although the exact role of gut microbiome in the pathogenesis of NAFLD remains unclear, there is a characteristic microbiome profile observed in NAFLD patients, with lower diversity and increased proportion of Coprococcus, Ruminococcus, Proteobacteria, and Enterobacteriaceae spp. NASH patients with advanced fibrosis, compared to those with or without early-stage fibrosis, had a higher proportion of Proteobacteria and *E. coli*, with a lower proportion of Firmicutes, especially *F. prausnitzii*^[118,119]^. Conflicting with their pathogenic and clinical significance, data regarding the qualitative and quantitative composition of intestinal microbiota have not yet entered the clinical arena.

## CONCLUSION

From a conceptual perspective, the NAFLD/NASH nosography continues to offer the advantages of precisely ruling out competing causes of liver disease (e.g., alcohol, viral infection, and others) and accurately describing liver histology changes. However, these do not necessarily need to be ruled out and reported in MAFLD diagnosis. MAFLD, on the other hand, probably offers the advantage of more accurately identifying the risk of target organ dysfunction, namely, progressive liver disease^[120]^, diabetes and chronic kidney disease^[121]^, atherosclerosis^[122]^, more severely impaired lung function^[123]^, colon cancer^[124]^, both intrahepatic and extrahepatic events^[125]^, and mortality^[126]^, although the last outcome is controversial^[127]^.

With this evolving scenario, the identification of cofactors for organ dysfunction^[128]^, which may contribute to explaining disease heterogeneity and consistently support inherently personalized medicine approaches, has been suggested as a possible solution to overcome the issue of non-responders to conventional therapeutic approaches in metabolic disorders and failures of NASH therapeutic trials^[9,129,130]^. To this end, an ever-increasing awareness of the type, number, and significance of NAFLD/NASH and MAFLD cofactors is a research priority, which opens the way to innovative pathogenic treatment strategies in this field.

## Figures and Tables

**Table 1.** Secondary NAFLD forms

Etiology	Comment	Authors
Viral-HCV	There are two different types of steatosis owing to HCV infection. HCV genotype 3 is directly steatogenic and has steatosis which is more common and consistent, whereas HCV genotypes other than genotype 3 exhibit lower prevalence and severity of steatosis, which is associated with the host’s metabolic determinants	Adinolfi *et al*.^[14]^
	HCV steatosis occurs in the setting of a complex pattern of metabolic alterations named “hepatitis C-associated dysmetabolic syndrome” (HCADS) also featuring hepatic steatosis; visceral fat hypertrophy; acquired, reversible hypocholesterolemia; and insulin resistance	Lonardo *et al*.^[15]^
	Strictly speaking, HCV-related steatosis cannot be classified as NAFLD and should best be named “MAFLD”	Polyzos *et al*.^[16]^
	HIV infection is strongly associated with steatosis. Formerly defined as “VAFLD” (virus-associated fatty liver disease), this entity should presently best be renamed “MAFLD”	Guaraldi *et al*.^[17]^ Liu *et al*.^[18]^
Viral-HIV		
Nutritional/intestinal-related causes	A variety of medico-surgical conditions, including acute weight loss (bariatric surgery and fasting), malnutrition, total parenteral nutrition, short bowel syndrome, intestinal failure, small intestinal bacterial overgrowth, microbiome changes, coeliac disease, and pancreatectomy, may lead to secondary NAFLD forms, some of which are highly progressive to cirrhosis	Liebe *et al*.^[13]^
		Angulo *et al*.^[19]^
Endocrine NAFLD/NASH	Polycystic ovary syndrome (PCOS), hypothyroidism, hypogonadism, and GH deficiency may be conceptualized as a naturally occurring disease model of NAFLD, which have specific pathomechanisms and are potentially reversible with specific treatment	Lonardo *et al*.^[20]^
Associated with pregnancy	Acute fatty liver of pregnancy	Azzaroli *et al*.^[21]^
Associated with metals and synthetic chemicals	Metals (such as lead) have been implicated in more fibrotic NAFLD forms in the NHANES population	Reja *et al*.^[22]^
	Environmental chemicals of industrial, agricultural, residential, and pharmaceutical origin can disrupt endocrine-metabolic pathways leading to secondary NAFLD forms	Heindel *et al*.^[23]^ Cano *et al*.^[24]^
Genetic disorders of metabolism	A variety of common and rare inherited metabolic disorders such as hemochromatosis, alpha-1 antitrypsin deficiency, Wilson’s disease, congenital lipodystrophy, glycogen storage diseases, hereditary fructose intolerance, urea cycle disorders, and citrullinaemia type 2 are associated with secondary NAFLD forms or worsen primary NAFLD	Liebe *et al*.^[13]^ Angulo *et al*.^[19]^
Drug-related	Many drugs can be steatogenic, including antiretrovirals, tamoxifen, corticosteroids, tetracyclines, valproic acid, amphetamines, and acetylsalicylic acid. However, drug-induced liver injury (DILI) is a definite disease entity other than NAFLD	Lammert *et al*.^[25]^
	Additionally, NAFLD patients may be at high risk of developing DILI, demonstrating that these are two different disease entities with some shared pathogenic aspects	Tarantino *et al*.^[26]^
